# SIns: A Novel Insertion Detection Approach Based on Soft-Clipped Reads

**DOI:** 10.3389/fgene.2021.665812

**Published:** 2021-04-30

**Authors:** Chaokun Yan, Junyi He, Junwei Luo, Jianlin Wang, Ge Zhang, Huimin Luo

**Affiliations:** ^1^School of Computer and Information Engineering, Henan University, Kaifeng, China; ^2^College of Computer Science and Technology, Henan Polytechnic University, Jiaozuo, China

**Keywords:** structural variation, alignment, short read, the next generation sequencing technology, soft-clipped read

## Abstract

As a common type of structural variation, an insertion refers to the addition of a DNA sequence into an individual genome and is usually associated with some inherited diseases. In recent years, many methods have been proposed for detecting insertions. However, the accurate calling of insertions is also a challenging task. In this study, we propose a novel insertion detection approach based on soft-clipped reads, which is called SIns. First, based on the alignments between paired reads and the reference genome, SIns extracts breakpoints from soft-clipped reads and determines insertion locations. The insert size information about paired reads is then further clustered to determine the genotype, and SIns subsequently adopts Minia to assemble the insertion sequences. Experimental results show that SIns can achieve better performance than other methods in terms of the F-score value for simulated and true datasets.

## Introduction

Although single-nucleotide polymorphisms (SNPs) represent the most frequent genomic variation, it is generally acknowledged that human genomes show more differences as a consequence of structural variations (SVs) (Gusnanto et al., 2012). SVs generally refer to genome sequence changes greater than 50 bp and can be further categorized as insertions, deletions, duplications, inversions, and translocations, among others, as well as combinations of these categories (Feuk et al., 2006; Alkan et al., 2011; Baker, 2012). Some studies have shown that phenotypic changes and some diseases are caused by SVs, e.g., autism, Parkinson’s disease, and schizophrenia (Suzuki et al., 2011). Therefore, the accurate detection of SVs is of great significance for gene expression analysis and related disease research (MacConaill and Garraway, 2010). However, until a few years ago, there were no efficient methods for the detection of SVs with high precision. The development of next-generation sequencing (NGS) technology has allowed researchers to obtain a large amount of sequence data, which has improved research on SV detection (The 1000 Genomes Project Consortium, 2010; Zhang et al., 2010; Guan and Sung, 2016; Kosugi et al., 2019).

As one type of SV, an insertion refers to the addition of a DNA sequence to the genome. This sequence might be novel or could exist in the original genome, which would be equivalent to translocation or duplication. In general, insertions can be divided into two types: (i) novel insertions refer to the insertion of a sequence that cannot be found or mapped to the reference genome, and (ii) mobile element insertions or duplications constitute insertions in which the sequence comes from the original sequence. The sequence of this second type of insertion can be obtained through a comparison with the reference genome. Based on the identification of discordant patterns in sequence data, some SV detection methods can currently be utilized to detect insertions. In general, these methods can be categorized into the following four classes: (i) paired-end mapping (PEM-based methods, such as BreakDancer (Chen et al., 2009), PEMer (Korbel et al., 2009) and GASV (Sindi et al., 2009)), which is based on the physical position and distance information of paired-end or mate-pair reads (Lee et al., 2009; Hormozdiari et al., 2010); (ii) split read (SR)-based methods, which search for split alignments of unmapped or clipped reads, and an example is CREST, which uses clipped reads to identify structural variations through multiple alignments and assembly (Wang et al., 2011); (iii) depth of coverage (DoC)-based methods such as SegSeq (Chiang et al., 2009), EWT (Yoon et al., 2009) and CNVnator (Abyzov et al., 2011)), which provide a macroscopic view of whether there is a high coverage area on the genome; and (iv) *de novo* assembly, which uses related reads to recover insertion sequences. The latter methods, such as ANISE and BASIL (Holtgrewe et al., 2015), SvABA (Wala et al., 2018), EPGA (Luo et al., 2015b) and EPGA2 (Luo et al., 2015a), require a coverage depth that is not less than 40X and have a high cost. However, these methods usually focus on abnormal information, such as variations in the insertion size and soft-clipped information, and thus cannot yield accurate detection results for insertions with variable sizes.

Some hybrid methods have been proposed for the detection of insertions with variable sizes in recent years. For example, Pindel, as a classical method, is mainly designed for deletions and small insertions and uses PEM and SR signatures to locate the breakpoints (Ye et al., 2009). However, for large insertions over 50 bp, Pindel does not perform well and yields many false positive results. MindTheGap uses a k-mer-based method to detect the insertion site and recovers insertion sequences through an assembly of k-mers (Rizk et al., 2014). This method enables the detection of small and large insertions, but the methods finds it difficult to locate a breakpoint when other polymorphisms occur near the insertion site, which leads to a high number of false negative results. As an insertion detection approach based on breakpoints, BreakSeek applies a Bayesian model for the PEM and SR signatures to find the accurate position of an insertion (Zhao and Zhao, 2015). The BreakSeek method can obtain accurate breakpoint results and genotypes without assembly, but the coverage depth of the dataset has some impact on the performance. In addition, although some insertion detection methods, such as PopIns (Kehr et al., 2016) and Pamir (Kavak et al., 2017), perform well, they may require a large number of data points.

In this paper, we propose an insertion detection approach called SIns, which is based on soft-clipped reads and achieves high insertion detection accuracy. SIns adopts PEM to identify and correct the breakpoints from a previous analysis of soft-clipped reads and clusters the insert size to determine the genotype. For sequence assembly, SIns directly extracts all abnormal reads and uses Minia to recover the insertion sequences. We conducted experiments using simulated data and real datasets, and the results show that SIns exhibits high accuracy in breakpoint detection and genotype determination.

The rest of this paper is organized as follows: in Section 2, we introduce the proposed method in detail. The experimental results are shown in Section 3, and we summarize and discuss the findings in Section 4.

## Methods

**FIGURE 1:**
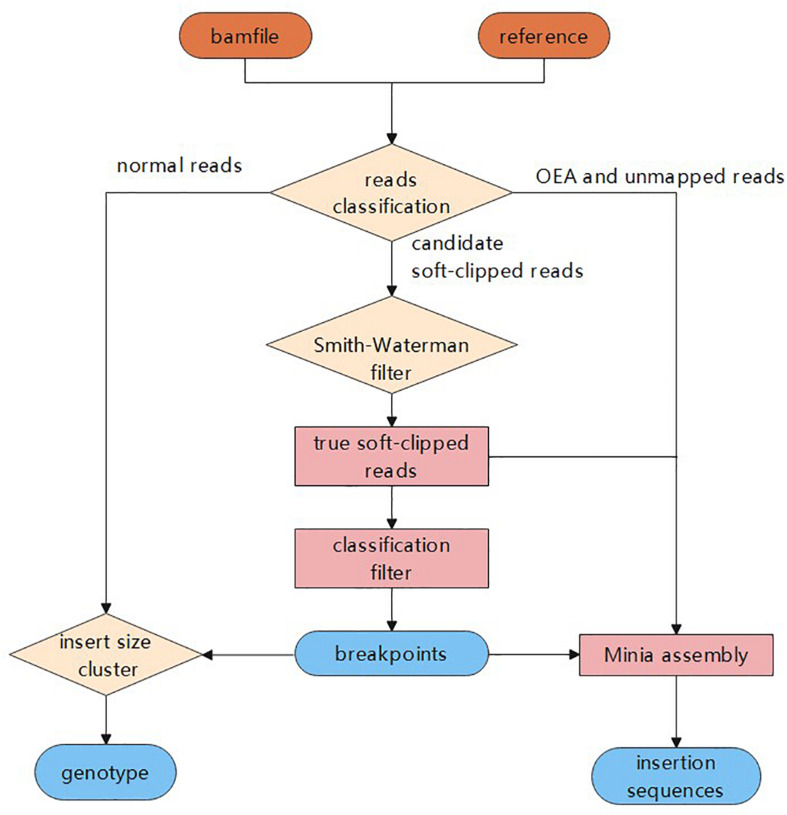
The process of Sins.

In this study, we propose a novel insertion detection approach named SIns for the detection of insertions based on soft-clipped reads. In general, SIns performs the following three steps: (i) breakpoint detection, determining the location of insertions based on comprehensive information; (ii) genotyping, identifying the genotype of the insertion based on clustering results; and (iii) assembly of insertion sequences. The overall pipeline of SIns is shown (Figure 1).

### Breakpoint Detection

Breakpoint detection is an important step in SIns. In this study, the breakpoints can be obtained through the following steps.

#### Step 1 Selection of Soft-Clipped Reads

For each soft-clipped read, SIns first obtains its clipped part, S_c_, and then extracts a sequence S_r_ from the reference genome, which corresponds to S_r_. Note that the length of S_r_ equals that of S_c_.

Based on the Smith-Waterman algorithm, a score matrix between S_c_ and S_r_ can then be constructed to reflect their detailed matching degree. Moreover, SIns can obtain the maximum score from the matrix, which refers to the length of the longest successive sequence. To identify and screen out real soft-clipped reads, a threshold parameter c is then set to select those reads whose S_c_ and S_r_ exhibit higher similarity. This parameter c can be computed using the following equation:

(1) $$c=\left\{ \begin{array}{c} 1,\max s\,c\,o\,r\,e<c\,l\,i\,p\,l\,e\,n\,g\,t\,h*m\\ 0,\max s\,c\,o\,r\,e\geq c\,l\,i\,p\,l\,e\,n\,g\,t\,h*m \end{array}\right.$$

where m represents the mappability (m ∈ [0,1]). If c equals 1, SIns selects it for the following steps; otherwise, SIns abandons it. A larger m indicates greater similarity between Sc and Sr. The default value for the parameter m is 0.5.

#### Step 2 Determination of Candidate Breakpoints

**FIGURE 2:**
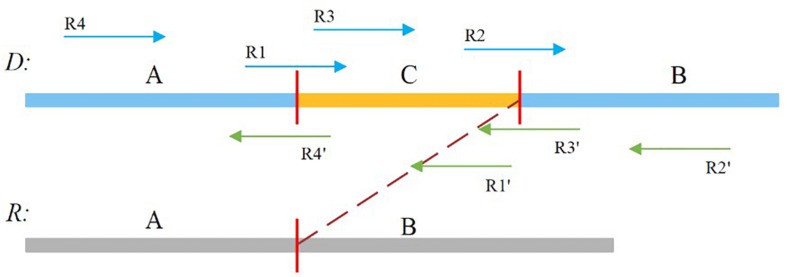
Sequence A and B is normal, and sequence C is insertion sequence. R1, R2, R4′,and R3′ are soft-clipped reads. R1 belongs to the LL type, R2 belongs to the LR type, R4′ belongs to the RL type, and R3′ belongs to the RR type.

In our study, the soft-clipped reads were further divided into four types, namely, LL, LR, RL, and RR, which are shown in Figure 2. Taking “LL” as an example, the first L means that the left mate read is soft-clipped, and the second “L” specifies that this read is clipped on its head, whereas “RR” indicates that the right mate read is soft-clipped on its tail.

A true insertion might be related to the four types of soft-clipped reads. These soft-clipped reads can provide similar breakpoint information. In general, an insertion breakpoint is regarded strongly as true if the four types of soft-clipped reads mentioned above exist. However, it is difficult to find all types of soft-clipped reads for a true insertion, particularly if the DoC is low. In this paper, SIns defines four types of breakpoints, which are represented as {LL, LR}, {LL, RL}, {RL, LR}, and {RL, RR}. For a breakpoint, SIns collects all related soft-clipped reads that are kept to PSD and determines their types, and SIns then uses the following equation to determine whether a breakpoint is true:

(2) J=(L⁢L∨R⁢L)⁢∧⁢(L⁢R∨R⁢R)

where LL∧LR indicates that the PSD of a breakpoint contains LL and LR, and LL∨RL indicates that it contains LL or RL. Subsequently, SIns obtains a list of breakpoints using the above-described method. However, the method yields some false positive breakpoints, which can be due to a high GC content, sequencing error or SNPs. Therefore, even though their proportion is small, these breakpoints should be checked and filtered.

#### Step 3 Filtering of the Breakpoints

Through the above-described steps, SIns can obtain candidate breakpoints, which might include some false breakpoints. SIns then uses a filter method based on the insertion size to further improve the precision of these breakpoints. An insertion usually causes a series of abnormal reads with an anomalous insert size distribution.

**FIGURE 3:**
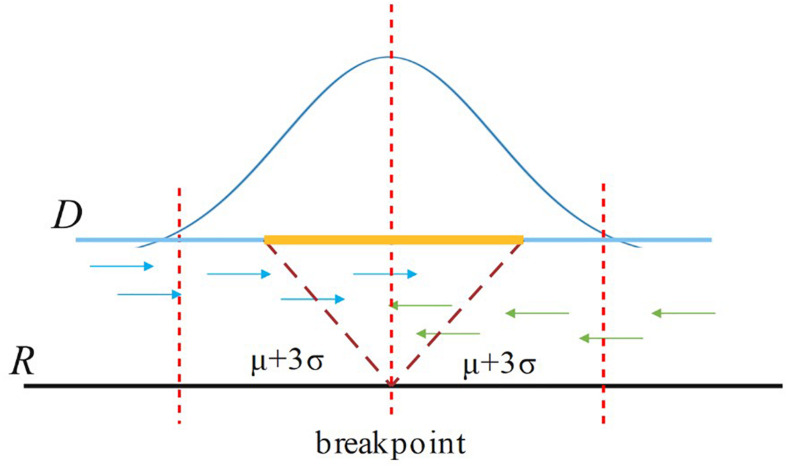
For a breakpoint, SIns only consider reads aligned in the region [p – (μ+3σ), p + (μ+3σ)], where p is the position of the breakpoint.

For a candidate breakpoint, SIns first finds the paired reads that span this breakpoint and OEA reads (one-end-anchored reads). Note that these reads should be aligned in the region [p − (μ++3σ), p + (μ+3σ)], where p is the position of the breakpoint, μ is the insert size of the read library, and σ is the standard deviation of μ as shown in Figure 3. If the sum of paired reads and OEA reads is larger than Cov/2, SIns treats this breakpoint as true, otherwise, the method considers the breakpoint to be false. Cov is the coverage of the read library.

### Genotyping

Genotyping is a necessary step of SIns. In a polyploid, the genotype is divided into heterozygous and homozygous genotypes. Taking diploid as an example, a heterozygous variation is only included in one chromosome and not the other one contains. In contrast, homozygosity indicates that the same variation is found in both chromosomes.

Genotyping can provide great convenience for subsequent studies, and many approaches, particularly assembly-based methods, are available for genotyping; however, all the assembly-based methods usually require considerable time and memory. Here, SIns adopts a cluster-based method, which can save as much time as possible.

**FIGURE 4:**
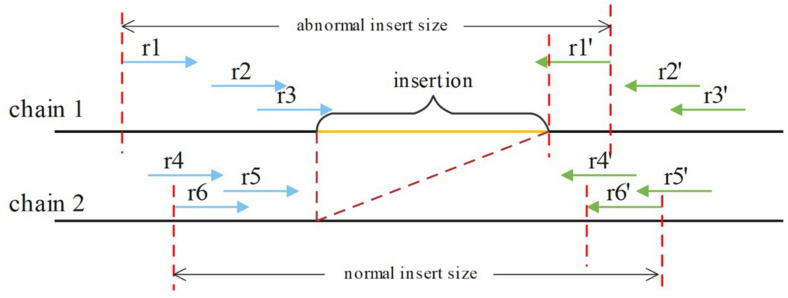
The paired reads (r1, r1′), (r2, r2′), and (r3, r3′) are obtained on the first chain, which contains an insertion. The other paired reads were obtained on the normal chain as shown. These insert sizes can be clustered into two clusters.

If an insertion occurs, it will inevitably cause a change in the insert size for paired reads around the breakpoint, such as OEA reads, and a decrease in the normal insert size. For a heterozygous insertion, the insert size is difficult to determine because the paired reads might originate from two different chromosomes. Some paired reads contain insertions, whereas others do not. We defined *P* (*P_*l*_, P_*r*_, and i*) for a paired read spanning the breakpoint, where *P*_*l*_ is the aligning position of the left mate read, *P*_*r*_ is the aligning position of the right mate read and *i* is the insert size value around this paired read. After obtaining P for all paired reads spanning the breakpoint, SIns applies the DBSCAN for clustering. In DBSCAN, the parameter eps = 50, min_samples = 2 in default, and these parameters can be adjusted. And, SIns determines a breakpoint as heterozygote if there is one cluster in the clustering result, otherwise, the breakpoint is deemed as homozygous. Two types of insert size distributions are shown in Figure 4.

### Assembly Insertion Sequences

In the assembly stage, SIns extracts OEA, soft-clipped and unmapped reads for a breakpoint to recover all possible insertion sequences. After applying the Minia (Boeva et al., 2012) algorithm to these abnormal reads, SIns generate a series of sequences with overlap, which contain insertion sequences. SIns then maps these sequences to the reference genome and obtains the insertion sequence results. For example, if the CIGAR value of a candidate sequence is 132M186I130M, the algorithm finds the length of this insertion, i.e., 186 bp, and determines that the sequence content is 133–318 bases.

## Experiments and Analysis

To verify the performance of SIns, we used SURVIVOR (Jeffares et al., 2017) and ART (Huang et al., 2012) to simulate a large number of insertions on human chromosome 22 ranging in size from 50 to 1,500 bp and in coverage from 5X to 50X. The recent popular detection methods MindTheGap and BreakSeek were compared with the proposed SIns method. In addition, the real human dataset NA12878 was selected to test the performance of SIns.

### Experimental Settings

#### Simulation Datasets and Parameter Setting

The simulation dataset was based on human chromosome 22, and the error rate of the dataset was set to 0.1%. SURVIVOR was used to simulate the structural variation. Here, we selected insertions for the simulation, and other types of structural variations were set to 0. ART was used to simulate different read sets from the simulated chromosome 22 containing insertions. We first generated some simulations of chromosome 22 containing insertions of different sizes, namely, 50–300 bp, 301–600 bp, 601–1,000 bp, and 1,001–1,500 bp, and ART was then used to simulate read sets with different coverages, i.e., 5X, 10X, 20X, 30X, 40X, and 50X. The read length was uniformly set to 150 bp, the inset size was 500 bp, and the standard deviation was 50. Using the above parameters, we can understand the detection ability of SIns under various conditions.

#### Evaluation Metrics

If the difference between the detected breakpoint and the simulated breakpoint does not exceed 10 bp, we consider it a positive result, which is represented by TP; otherwise, the result is represented by FP. True breakpoints that were not detected are indicated by FN. To clearly show the detection performance of various methods, we used the metrics precision (Pr), recall (Rc) and F-score as follows:

(3) $$P_{r}=\frac{T\,P}{T\,P+F\,P}\times 100\%$$

(4) $$R_{c}=\frac{T\,P}{T\,P+F\,N}\times 100\%$$

The F-score was defined as the harmonic average of precision and recall:

(5) $$F_{s\,c\,o\,r\,e}=\frac{2\,P_{r}\times R_{c}}{P_{r}+R_{c}}\times 100\%$$

### Simulation Dataset

#### Results on Homozygous Dataset

We compared SIns with MindTheGap and BreakSeek, selected chromosome 22 as the reference and simulated a chromosome containing 1,051 insertions of 50–300 bp, a chromosome containing 597 insertions of 301–600 bp, a chromosome containing 597 insertions of 601–1,000 bp and a chromosome containing 790 insertions of 1,001–1,500 bp. Based on different coverages, we simulated six read sets for each simulated chromosome. The experimental results are shown in Table 1.

**TABLE 1:** Comparison of three tools for four ranges.

Doc	Tool	50-300			301–600			601-1,000			1,001-1,500		
					
		Pr	Rc	F-score	Pr	Rc	F-score	Pr	Rc	F-score	Pr	Rc	F-score
5X	SIns	99.784	**87.726**	**93.367**	**100**	**64.992**	**78.782**	**100**	**61.977**	**76.525**	**100**	**63.924**	**77.992**
	BreakSeek	**99.791**	45.48	62.484	**100**	14.405	25.183	98.592	11.725	20.958	**100**	11.899	21.267
	MindTheGap	11.949	26.546	16.48	2.317	27.471	4.274	3.104	26.801	5.563	4.551	29.494	7.885
10X	SIns	99.412	**96.48**	**97.924**	99.815	**90.62**	**94.996**	**100**	**89.615**	**94.523**	**100**	**90.127**	**94.807**
	BreakSeek	**99.892**	87.631	93.36	**100**	61.809	76.398	99.701	55.946	71.674	99.774	55.823	71.591
	MindTheGap	30.356	64.986	41.381	20.918	65.662	31.728	21.315	67.337	32.38	25.962	67.468	37.496
20X	SIns	99.037	**97.812**	**98.42**	**99.65**	**95.477**	**97.519**	**100**	**93.802**	**96.802**	**99.868**	**95.57**	**97.671**
	BreakSeek	**99.603**	95.433	97.473	99.27	91.122	95.022	99.259	89.782	94.283	99.447	91.013	95.043
	MindTheGap	85.845	80.209	82.932	75.955	79.899	77.878	73.242	80.235	76.579	79.597	80	79.798
30X	SIns	**98.848**	**98.002**	**98.423**	**99.308**	**96.147**	**97.702**	**100**	**94.807**	**97.334**	**99.867**	**95.316**	**97.539**
	BreakSeek	99.509	96.384	97.922	99.298	94.807	97.001	99.284	92.965	96.021	99.459	93.165	96.209
	MindTheGap	86.829	81.541	84.102	77.564	81.072	79.279	75.425	81.742	78.457	80.73	81.139	80.934
40X	SIns	98.102	**98.382**	98.242	**100**	**96.482**	**98.21**	**99.825**	**95.477**	**97.603**	**99.868**	**95.949**	**97.87**
	BreakSeek	**99.708**	97.431	**98.556**	99.123	94.64	96.829	99.295	94.305	96.735	99.597	93.797	96.61
	MindTheGap	86.917	81.541	84.143	77.404	80.905	79.115	75.889	82.245	78.939	80.832	81.139	80.985
50X	SIns	98.57	**98.382**	98.476	**98.969**	**96.482**	**97.71**	**100**	**95.477**	**97.686**	**99.869**	**96.203**	**98.001**
	BreakSeek	**99.708**	97.431	**98.556**	98.614	95.31	96.934	99.118	94.137	96.564	99.338	94.937	97.087
	MindTheGap	87.018	81.637	84.242	77.28	80.905	79.051	75.153	82.077	78.463	80.881	81.392	81.136

*The bold values represent the highest value of each data set in different depth.*

As shown in Table 1, the performances of SIns and BreakSeek in detecting insertions of 50–300 bp were better. Although the precision of BreakSeek was generally higher than that of SIns, its F-score was only better than that of SIns when the coverages of the read set were 40X and 50X. We also found that SIns has a higher recall, which means that SIns can detect more true insertions. SIns exhibited higher precision and recall regardless of the coverage and the length of insertions. In addition, none of the methods worked well with low DoCs. However, for the case with a low coverage (DoC ≤ 10X), SIns showed better performance than the other methods.

#### Results on Heterozygous Dataset

To verify the performance of SIns in detecting heterozygous insertions, we simulated read sets of chromosome 22. Simulations of chromosome 22 containing insertions of 50–300 bp were used to produce these read sets, and other simulations of chromosome 22 containing an insertion of 301–600 bp were also used to generate other read sets. We then combine the read sets from the normal chromosome 22 and the simulations of chromosome 22. Note that the read sets were simulated with different coverages: 10X, 20X, 40X, 60X, and 80X. The experimental results are shown in Table 2.

**TABLE 2:** Result of 50–300 and 301–600 bp heterozygous insertions.

50-300	Tool	50-300			301-600		
			
		Pr	Rc	F-score	Pr	Rc	F-score
10X	SIns	**100**	**92.959**	**96.351**	**100**	**89.782**	**94.616**
	BreakSeek	**100**	33.111	49.75	**100**	21.441	35.31
	MindTheGap	11.275	21.789	14.86	5.211	22.111	8.435
20X	SIns	99.903	**97.907**	**98.895**	**100**	**96.985**	**98.469**
	BreakSeek	**99.707**	64.7	78.477	**100**	48.576	65.389
	MindTheGap	88.596	57.659	69.856	79.669	56.449	66.078
40X	SIns	**99.807**	**98.573**	**99.186**	**100**	**97.99**	**98.985**
	BreakSeek	98.847	65.271	78.625	98.805	41.541	58.491
	MindTheGap	98.609	67.46	80.113	97.387	68.677	80.55
60X	SIns	**99.425**	**98.763**	**99.093**	**100**	**97.99**	**98.985**
	BreakSeek	98.389	63.939	77.509	98.214	46.064	62.714
	MindTheGap	99.349	72.598	83.892	98.42	73.032	83.846
80X	SIns	**99.616**	**98.858**	**99.236**	**100**	**97.99**	**98.985**
	BreakSeek	98.503	62.607	76.556	98.264	47.404	63.955
	MindTheGap	98.84	72.978	83.963	98.42	73.032	83.846

*The bold values represent the highest value of each data set in different depth.*

As illustrated in Table 2, the detection results obtained with MindTheGap were less effective than those obtained with homozygous detection because MindTheGap has more sequences to choose from when selecting k-mers, which will yield some conflicting issues. The performance of BreakSeek on these two datasets was not as good as the results obtained with homozygotes, and a reason for this finding might be that normal reads extracted from the reference genome, which contained many contradictory PEM and SR information, were added. When BreakSeek iteratively analyses the PEM signature, there is too much contradictory information that can be used, and thus, the result cannot show the most authentic SV information. In contrast, when SIns extracts breakpoint information at the initial stage, the method relies more on SR information and thus experiences less interference from contradictory information. At the subsequent filtering stage, due to the addition of normal reads, the filtering conditions were more rigorous and precise, which explains why the precision of SIns increased, whereas the recall value decreased.

### Experiments Based on Real Dataset

NA12878 is the gold standard dataset commonly used in genomics. Experiments with NA12878 (ERR194147 50X^1^) samples were conducted using the SIns, MindTheGap and BreakSeek methods. We extracted the reads with a probability of 0.1 because the coverage was too high. The generally recognized VCF file of this sample contains 50,016 insertion reports larger than 50 bp. The corresponding vcf file can be downloaded from NCBI. We only selected the detected results in the file records as true values. The test results are shown in Table 3.

**TABLE 3:** Results obtained with NA12878.

	SIns	MindTheGap	BreakSeek
chr1	123	98	90
chr2	180	136	74
chr3	107	57	38
chr4	105	87	37
chr5	94	68	44
chr6	117	84	43
chr7	134	91	44
chr8	73	72	43
chr9	77	69	48
chr10	101	62	42
chr11	88	46	41
chr12	99	65	46
chr13	66	27	36
chr14	51	29	28
chr15	42	44	29
chr16	88	63	69
chr17	67	46	29
chr18	72	42	27
chr19	67	46	23
chr20	38	50	25
chr21	57	16	24
chr22	28	27	21

We have filtered out the SNPs and Indels of this data set. The above results show that SIns has good performance on most chromosomes compared with MindTheGap and BreakSeek. Although the detection number of insertions on chromosome 15 and 20 are lower than that of MindTheGap, we can find the result on the rest of chromosomes are better than other two methods. And the average of F-score on all 22 chromosomes is 5.46% for SIns. MindTheGap is 2.42%, and BreakSeek is 2.85%. The average of F-score shows the same conclusion.

### Running Time Comparison

Here we list the time comparison results of homozygote and heterozygous experiments.

Although clustering is useful in the SIns process, it does not require as many iterations as in BreakSeek, MindTheGap and other methods; thus, SIns exhibits a relatively obvious advantage in terms of running time. As shown in Tables 4, 5, all the methods were run in the same machine and a single thread by default. As a result, SIns exhibited better performance than the other two methods in most cases. The main time-consuming step of SIns is the third step: the reads used for assembly are extracted from the original read collection, which is the most work-intensive step. If the assembly is not considered and the method aims to just detect breakpoints and judge genotypes, SIns can complete the task within a short time.

**TABLE 4:** Homozygote results obtained with four ranges.

*Doc*	50–300			301–600			601–1,000			1,001–1,500		
				
	Mind TheGap	Break Seek	SIns	Mind TheGap	Break Seek	SIns	Mind TheGap	Break Seek	SIns	Mind TheGap	Break Seek	SIns
*5X*	176s	1868s	20s	174s	1842s	35s	178s	2130s	29s	177s	2127s	31s
*10X*	217s	1868s	40s	216s	2250s	68s	227s	2156s	65s	212s	2089s	61s
*20X*	243s	2177s	77s	242s	2178s	119s	242s	2054s	142s	235s	4349s	123s
*30X*	264s	2249s	116s	264s	2109s	180s	257s	3723s	191s	203s	5281s	184s
*40X*	284s	2415s	154s	286s	2589s	250s	292s	4948s	240s	204s	2736s	245s
*50X*	304s	2577s	193s	310s	2943s	343s	310s	3207s	319s	211s	2539s	307s

**TABLE 5:** Heterozygous results obtained with four ranges.

*Doc*	50–300			301–600		
		
	Mind TheGap	Break Seek	SIns	Mind TheGap	Break Seek	SIns
*10X*	140s	1997s	38s	139s	2020s	19s
*20X*	152s	2041s	76s	154s	1990s	47s
*40X*	171s	2224s	150s	180s	2495s	84s
*60X*	190s	2779s	227s	193s	2869s	122s
*80X*	212s	2703s	305s	215s	3294s	204s
*100X*	227s	3634s	425s	254s	3719s	259s

## Discussion

In this article, we propose an insertion detection method named SIns based on the comprehensive processing of soft-clipped read information. SIns can provide more precise detection of breakpoints and can perform relatively accurate genotyping. In addition, SIns uses the Minia algorithm for assembly of the insertion sequence, and the successfully assembled sequence is then filtered and tailored according to the breakpoint information. After these steps, the complete insertion sequence is provided.

Most of the existing methods show effectiveness in detecting small insertions but show poor performance in cases of low coverage. These methods usually are difficult to detect all types of SVs of all sizes. SIns focuses on the detection of insertions of different sizes. We tested the detection performance of SIns using various simulated datasets and compared it with MindTheGap and BreakSeek. In most cases, the performance of SIns was better than those of the other two methods. Comparing with the other two methods, SIns performs well both on low and high coverage data sets and different size insertions. The experimental results using a real dataset show that SIns exhibits good detection capability.

## Data Availability Statement

The datasets presented in this study can be found in online repositories. The names of the repository/repositories and accession number(s) can be found below: http://www.ebi.ac.uk/ena.

## Author Contributions

CY and JL conceived and designed the approach. JH performed the experiments. JW and GZ analyzed the data. JH and JL wrote the manuscript. JL and HL supervised the whole study process and revised the manuscript. All authors have read and approved the final version of manuscript.

## Conflict of Interest

The authors declare that the research was conducted in the absence of any commercial or financial relationships that could be construed as a potential conflict of interest.

## Footnotes

1 http://www.ebi.ac.uk/ena
